# *Ralstonia solanacearum* Acetyltransferase RipU Hijacks SlJAR1 to Inhibit Jasmonic Acid Signalling and Facilitate Pathogen Infection

**DOI:** 10.1111/pbi.70522

**Published:** 2026-01-12

**Authors:** Tong Qin, Shen Cong, Xiuan Liang, Fenglei Li, Xiaoyan Liang, Zhiheng Zhang, Yongqiang He, Shanshan Yang, Xiaoxiao Zhang, Hai‐Lei Wei

**Affiliations:** ^1^ Guangxi Key Laboratory of Agro‐Environment and Agro‐Product Safety, College of Agriculture Guangxi University Nanning China; ^2^ State Key Laboratory of Efficient Utilization of Arable Land in China, Institute of Agricultural Resources and Regional Planning Chinese Academy of Agricultural Sciences Beijing China

**Keywords:** hormone modulation, plant immunity, protein acetylation, *Ralstonia solanacearum*, RipU, SlJAR1, Type III effector

1

*Ralstonia solanacearum* delivers numerous Type III effectors (T3Es) to cause bacterial wilt. These T3Es serve as molecular probes for identifying plant immunity components (Islam et al. 2024). We previously identified the T3E RipU from *R. solanacearum* strain P380. When delivered by a T3E‐deficient *Pseudomonas* mutant D36E, RipU suppressed pathogen‐associated molecular patterns (PAMPs)‐triggered immunity (PTI) and induced cell death in *Nicotiana benthamiana* (Cong et al. 2023). However, whether RipU regulates immunity in tomato, the natural host of strain P380, remains unknown.

To investigate the function of RipU in tomato, we infiltrated leaves with D36E harbouring empty vector (EV) or RipU. RipU suppressed reactive oxygen species (ROS) burst and induced cell death with increased ion leakage compared to EV (Figure 1a–c). Moreover, *ripU*‐overexpressing (OE‐RipU) tomato plants exhibited suppressed flg22‐induced ROS production and decreased resistance to both *R. solanacearum* P380 and *Pst* DC3000 (Figure 1d, Figure S1a–c). Soil‐drench inoculation showed that the *ripU* mutant attenuated virulence in tomato and *Arabidopsis thaliana* , which was restored by complementation (Figure S2a–d). Consistently, the mutant displayed impaired bacterial growth in *A. thaliana* , a defect also restored upon complementation (Figure S2e). These results demonstrate that RipU suppresses tomato immunity and is essential for *R. solanacearum* virulence.

**FIGURE 1:**
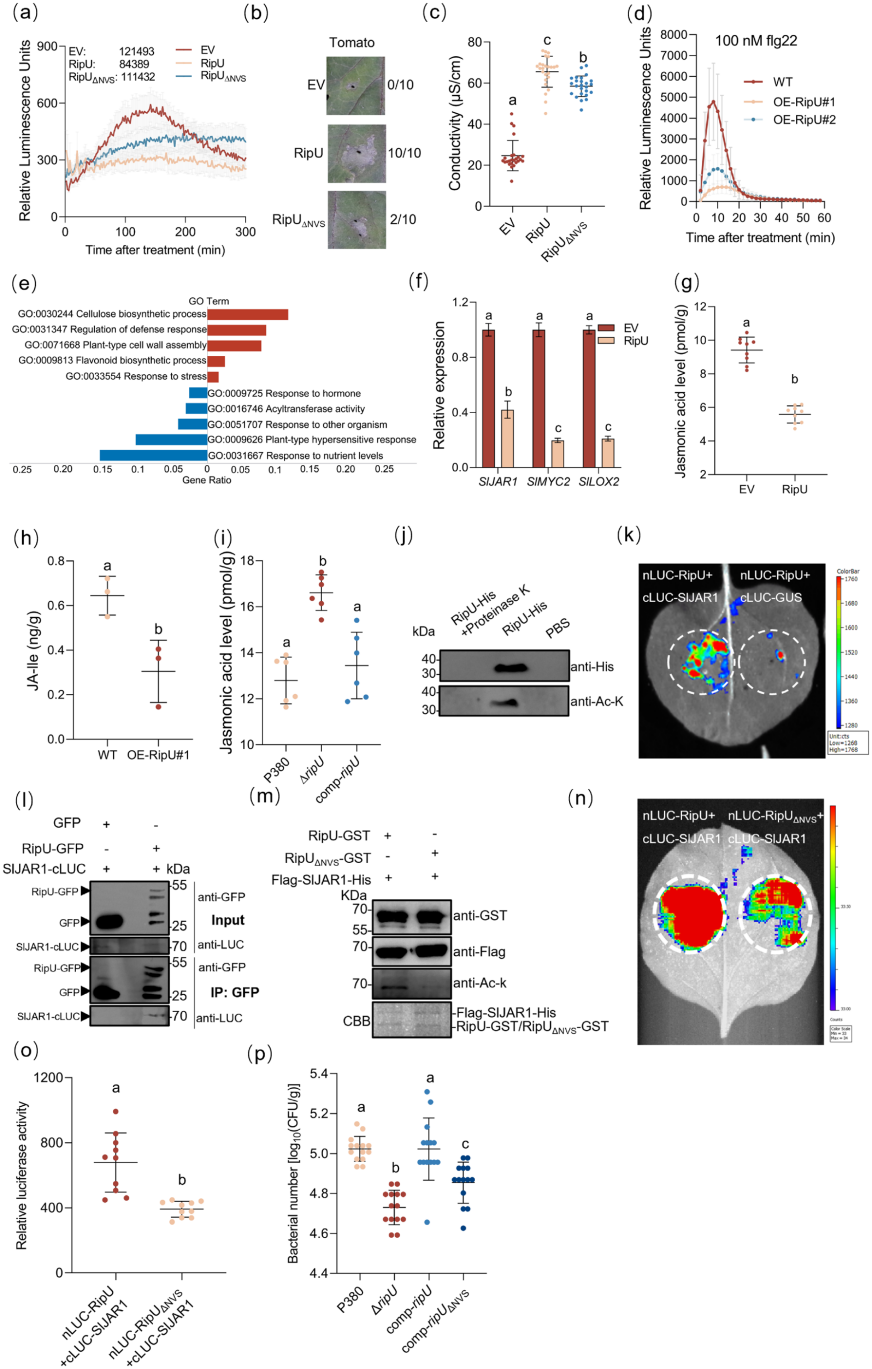
RipU interacts with SlJAR1 to promote infection. (a) ROS production in tomato leaves infiltrated with D36E and derivatives (6 × 10^8^ CFU/mL) at 6 hpi (*n* = 12). (b) Cell death in tomato leaves infiltrated with D36E and derivatives (10^8^ CFU/mL), imaged at 24 hpi (*n* = 10). (c) Conductivity assay in tomato leaves infiltrated with D36E and derivatives (6 × 10^8^ CFU/mL), sampled at 12 hpi (*n* = 25). (d) flg22‐induced ROS production in tomato leaves (*n* = 12). (e) GO enrichment of RipU‐induced DEGs; red and blue denote up‐ and downregulated genes, respectively. (f) Expression of JA‐related genes in tomato leaves infiltrated with D36E and derivatives (6 × 10^8^ CFU/mL), sampled at 6 hpi (*n* = 9). (g) JA content in tomato leaves infiltrated with D36E and derivatives (6 × 10^7^ CFU/mL), sampled at 24 hpi (*n* = 9). (h) JA‐Ile content in roots of WT or OE‐RipU tomato plants (*n* = 3). (i) JA levels in tomato roots at 48 hpi following dip inoculation of tomato with *R. solanacearum* P380 and derivatives (10^8^ CFU/mL; *n* = 6). (j) Autoacetylation of RipU detected by immunoblot with anti‐His and anti–Ac‐K antibodies; RipU treated with Proteinase K and PBS solution were used as controls. (k) Split‐LUC and (l) Co‐IP assays showing RipU–SlJAR1 interaction in *N. benthamiana* leaves. (m) RipU acetylation of SlJAR1 in *E. coli* co‐expressing RipU‐GST (or RipU_ΔNVS_‐GST) and Flag‐SlJAR1‐His, detected by anti–Ac‐K immunoblot; CBB staining confirmed equal loading. (n, o) Split‐LUC assays show the NVS motif is essential for RipU–SlJAR1 interaction (*n* = 10). (p) Bacterial population in stems of tomato plants root‐inoculated with P380 and derivatives (10^8^ CFU/mL), measured at 3 dpi (*n* = 14). Data are mean ± SD; different lowercase letters indicate significant differences (*p* < 0.05, one‐way ANOVA).

We performed RNA sequencing on tomato leaves infiltrated with D36E harbouring EV or RipU to characterise the transcriptional defence response. The six RNA‐seq libraries generated approximately 263 million reads with a 97% genome mapping rate. RipU treatment upregulated 2704 genes (7.94% of genome) and downregulated 4404 genes (12.92% of genome) compared to EV (≥ 2‐fold change, *p* < 0.05; Figure S3a). Differentially expressed genes (DEGs) analysis revealed that a set of immune‐associated genes was downregulated by RipU (Figure S3b,c). Additionally, both Gene Ontology (GO) and Kyoto Encyclopedia of Genes and Genomes (KEGG) analyses showed significant enrichments in plant hormone response among downregulated genes (Figure 1e, Figure S3d,e). We next investigated whether RipU altered the expression of genes associated with jasmonic acid (JA), a plant hormone that mediates resistance against *R. solanacearum* (Jiang et al. 2025). Reverse transcription quantitative PCR (RT‐qPCR) revealed that RipU inhibited the expression of *SlJAR1*, *SlMYC2* and *SlLOX2* (Figure 1f). Consistent with this, RipU significantly reduced endogenous JA levels in tomato leaves compared to EV (Figure 1g). Furthermore, overexpression of *ripU* significantly decreased levels of JA‐Isoleucine (JA‐Ile), a key bioactive jasmonate synthesised by SlJAR1, in tomato roots (Figure 1h) (Suza et al. 2010). We next examined whether RipU altered the levels of JA in tomato roots during pathogen infection. The Δ*ripU* mutant inoculation resulted in greater JA accumulation than the wild‐type (WT) P380 strain. This effect was reversed by complementation with *ripU* (comp‐*ripU*) (Figure 1i). These results suggest that RipU inhibits JA accumulation.

Given that many T3Es inducing cell death possess acetyltransferase activity (Ma and Ma 2016), we tested RipU in an in vitro assay and confirmed its autoacetylation activity (Figure 1j). However, RipU shows low sequence conservation with known acetyltransferase T3Es and lacks the conserved catalytic cysteine (Figure S4). Based on the central role of SlJAR1 in JA signalling (Suza et al. 2010), we hypothesized RipU targets it via acetylation. We confirmed the physical interaction between RipU and SlJAR1 *in planta* using split‐luciferase complementation (Split‐LUC) and co‐immunoprecipitation (Co‐IP) assays (Figure 1k,l). We next employed immunoblotting with anti‐acetyl‐lysine (Ac‐K) antibody to investigate whether RipU could acetylate SlJAR1. Acetylated SlJAR1‐Flag was detected with RipU‐GFP but not GFP alone (Figure S5). These results suggest that SlJAR1 may be an acetylation substrate of RipU. Our structural modelling of RipU indicated that the amino acids at positions 203–205 (NVS residues) are likely critical sites. We mutated these three amino acids to alanine to generate RipU_ΔNVS_ and assessed its activity. Immunoblot analysis with anti‐Ac‐K antibody showed that the acetylation level of Flag‐SlJAR1‐His was attenuated when co‐expressed with RipU_ΔNVS_‐GST compared to RipU‐GST in *Escherichia coli* cells (Figure 1m). These results indicate that RipU acetylates SlJAR1 partially depend on the NVS residues. Importantly, mutation of the NVS residues also impaired the interaction between RipU and SlJAR1 (Figure 1n,o). Furthermore, we investigated whether RipU contributed to *R. solanacearum* virulence via its acetyltransferase activity. Mutation of NVS residues in RipU compromised its ROS‐suppressive and cell death‐inducing activities in both *N. benthamiana* and tomato leaves (Figure 1a–c, Figure S6). We next quantified the bacterial populations of *R. solanacearum* in tomato xylem. In line with the established role of RipU in *R. solanacearum* pathogenesis (Hiles et al. 2024), we observed a significant reduction of the Δ*ripU* mutant in bacterial replication (Figure 1p). This reduction was fully rescued by comp‐*ripU* and only partially rescued by complementation with *ripU*_ΔNVS_ (comp‐*ripU*_ΔNVS_) (Figure 1p). These results indicate that the acetyltransferase activity of RipU is required for full bacterial virulence.

In summary, RipU is a unique T3E acetyltransferase that suppresses JA‐mediated immunity by targeting SlJAR1. It lacks homology and the catalytic cysteine of known T3E acetyltransferases, revealing a novel hormone‐sabotage strategy for bacterial wilt control.

## Ethics Statement

The authors have nothing to report.

## Conflicts of Interest

The authors declare no conflicts of interest.

## Supporting information


**Data S1:** pbi70522‐sup‐0001‐Supinfo.docx.

## Data Availability

All data supporting this study are included in the paper and its Supporting Information S1.
