# Supplementary material for: Ralstonia solanacearum Acetyltransferase RipU Hijacks SlJAR1 to Inhibit Jasmonic Acid Signalling and Facilitate Pathogen Infection
Source: Plant Biotechnol J. 2026 Jan 12;24(5):2914–6. doi: 10.1111/pbi.70522 (PMC13110135; doi:10.1111/pbi.70522)
Supplement: Supplementary file 1 — Data S1: pbi70522‐sup‐0001‐Supinfo.docx. [file PBI-24-2914-s001.docx]

***Ralstonia solanacearum* acetyltransferase RipU hijacks SlJAR1 to inhibit jasmonic acid signaling and facilitate pathogen infection**

Tong Qin^1#^, Shen Cong^2#^, Xiuan Liang^1#^, Fenglei Li^1^, Xiaoyan Liang^1^, Zhiheng Zhang^1^, Yongqiang He^1^, Shanshan Yang^1^, Xiaoxiao Zhang^1*^, Hai-Lei Wei^2*^

^1^Guangxi Key Laboratory of Agro-environment and Agro-product Safety, College of Agriculture, Guangxi University, Nanning 530004, China

^2^State Key Laboratory of Efficient Utilization of Arable Land in China, Institute of Agricultural Resources and Regional Planning, Chinese Academy of Agricultural Sciences, Beijing 100081, China

* Correspondence:

Prof. Hai-Lei Wei; Email: [weihailei@caas.cn](mailto:weihailei@caas.cn)

Dr. Xiaoxiao Zhang; Email: [zhangxiao0719@126.com](mailto:zhangxiao0719@126.com)

^#^ These authors contributed equally to this work.

| **Strains and plasmids used in this study** | | | |
| --- | --- | --- | --- |
| **Strains and plasmids** | **Characteristics** | | **Source** |
| **Strains** |  | |  |
| ***Escherichia coli*** |  | |  |
| *DH5α* | *supE44* *ΔlacU169* (*Φ80/lacZ*ΔM15)  *hsdR17 recA1 endA1 gyrA96*  *thi-1 relA1* | | TIANGEN |
| ***Agrobacterium*** |  | |  |
| GV3101 | *Agrobacterium tumefaciens* strain for plant transformation; Rif^R^, Gm^R^ | | TIANGEN |
| ***Pseudomonas syringae*** |  | |  |
| D36E | The derivative strain of *Pst* DC3000, with 36 effectors deleted, Rif^R^, Spe^R^ | | Wei et al., 2018. |
| D36E-EV | D36E containing pCPP5371 empty vector; Rif^R^, Spe^R^, Gm^R^ | | Cong et al., 2023. |
| D36E-RipU | D36E containing pCPP5371 carrying *ripU*; Rif^R^, Spe^R^, Gm^R^ | | Cong et al., 2023. |
| D36E-RipU_∆NVS_ | D36E containing pCPP5371 carrying *ripU_∆NVS_*; Rif^R^, Spe^R^, Gm^R^ | | This study |
| ***Ralstonia solanacearum*** |  | |  |
| P380 | Cm^R^ | | Cong et al., 2023. |
| Δ*ripU* | *ripU* markerless mutant of P380; Cm^R^ | | This study |
| *ripU*-comp | Δ*ripU* containing pBBRMCS2 carrying *ripU* with its native promoter; Cm^R^, Km^R^ | | This study |
| *ripU_Δnvs_*-comp | Δ*ripU* containing pBBRMCS2 carrying *ripU_Δnvs_* with its native promoter; Cm^R^, Km^R^ | | This study |
| **Plasmids** |  | |  |
| pK18mobsacB | Suicide vector with *sacB* gene; Km^R^ | | Lab collection |
| pK18*ripU* | Suicide vector containing upstream and downstream fragments of *ripU* gene on pK18mobsacB; Km^R^ | | This study |
| pBBRHB*ripU* | The full-length sequence of the *ripU* gene on the pBBR1MCS-2; Km^R^ | | This study |
| pBBRHB*ripU_ΔNVS_* | The full-length sequence of the *ripU_ΔNVS_* gene on the pBBR1MCS-2; Km^R^ | | This study |
| pGEX-6P-1-RipU | pGEX-6P-1 fused RipU | | This study |
| pET-30a-SlJAR1 | | pET-30a fused SlJAR1 | This study |
| pSuper1300-3×FLAG | | pSuper1300 fused 3×FLAG tag | Lab collection |
| pSuper 1300-SlJAR1 | | pSuper1300-3×FLAG fused SlJAR1 | This study |
| **Strains and plasmids** | | **Characteristics** | **Source** |
| pSuper1300-eGFP | | pSuper1300 fused eGFP tag | Lab collection |
| pSuper1300-eGFP-RipU | | pSuper1300-eGFP fused RipU | This study |
| pGWB554 | | Gateway cloning compatible binary vector with N-terminal fused mRFP (*CaMV35S* promoter); Spe^R^ | Lab collection |
| pGWB554-RipU | | pGWB554 fused RipU; Spe^R^ | This study |
| pGWB554-RipU_∆NVS_ | | pGWB554 fused RipU_∆NVS_; Spe^R^ | This study |
| pDONR221 | | A Gateway cloning vector for entry cloning; Km^R^ | Lab collection |
| pDONR221-RipU | | pDONR221 fused RipU; Km^R^ | This study |
| pDONR221-RipU_∆NVS_ | | pDONR221 fused RipU_∆NVS_; Km^R^ | This study |
| pCPP5371 | | A Gateway destination vector for effector protein expression driven by the *avrPto* promoter with a C-terminal Cya tag; Gm^R^ | Cong et al., 2023. |
| pCPP5371-RipU | | pCPP5371 fused RipU; Gm^R^ | Cong et al., 2023. |
| pCPP5371-RipU_∆NVS_ | | pCPP5371 fused RipU_∆NVS_; Gm^R^ | This study |
| pCAMBIA 1300-nLUC | | Plant transformation vectors carrying NanoLuciferase N-terminal fragment (nLUC); Km^R^ | Zhou et al., 2018. |
| pCAMBIA1300-cLUC | | Plant transformation vectors carrying the C-terminal fragment of NanoLuciferase (cLUC); Km^R^ |  |
| pCAMBIA1300-nLUC-RipU | | pCAMBIA 1300-nLUC fused RipU; Km^R^ | This study |
| pCAMBIA1300-nLUC-RipU_ΔNVS_ | | pCAMBIA 1300-nLUC fused RipU_ΔNVS_; Km^R^ | This study |
| pCAMBIA1300-cLUC-SlJAR1 | | pCAMBIA 1300-cLUC fused SlJAR1; Km^R^ | This study |
| pCAMBIA1300-cLUC-GUS | | pCAMBIA 1300-nLUC fused GUS; Km^R^ | This study |

| **Primers used in this study** | | |
| --- | --- | --- |
| **Primer name** | **Sequence (5’-3’)** | **Description** |
| RipU-attb1 | GGGGACAAGTTTGTACAAAAAAGCAGGCTATGAACCGCGTCGGGAAA | For cloning *ripU* and constructing it into the pDONR221 vector |
| RipU-attb2 | GGGGACCACTTTGTACAAGAAAGCTGGGTCTGCGGCGCGTCCTGG |  |
| superRipU-GFP-F | CTGCAGGGGCCCGGGGTCGACATGAACCGCGTCGGGAAAT | For cloning *ripU* and constructing it into the pSuper1300-GFP vector. |
| superRipU-GFP-R | GCCCTTGCTCACCATGGTACCCTGCGGCGCGTCCTGG |  |
| RipU-L1 | CTATGACATGATTACGAATTCACCGCCATCTCCATCCTG | For deleting the *ripU* gene, located upstream of the *ripU* gene. |
| RipU-L2 | CTAGCGCGACGGCTTACTGACGCGGTTCATGGTGGTTT |  |
| RipU-R1 | AAACCACCATGAACCGCGTCAGTAAGCCGTCGCGCTAG | For deleting the *ripU* gene, located downstream of the *ripU* gene. |
| RipU-R2 | CAGGTCGACTCTAGAGGATCCCCACGCCTCCACGCTGTAT |  |
| RipU-HBF | AGGGAACAAAAGCTGGGTACCATGAACCGCGTCGGGAAA | For cloning the *ripU* and *ripU*_ΔNVS_ genes full-length CDS and constructed the pBBRMCS2 vector. |
| RipU-HBR | TCCCCCGGGCTGCAGGAATTCTTACTGCGGCGCGTCC |  |
| RipU-JCF | GCCTTCACCGTCCTCTCT | Primers for detection of the *ripU* and *ripU*_ΔNVS_. |
| RipU-JCR | CAAAATCTTGCGGACGGA |  |

| **Primer name** | **Sequence (5’-3’)** | **Description** |
| --- | --- | --- |
| Kan-TF | GGCTATGACTGGGCACAACA | Primers for verification of the pBBR-MCS2 and pK18mobsacB vector. |
| Kan-TR | GCAGGAGCAAGGTGAGATGAC |  |
| nLUC-RipU-F | ACGGGGGACGAGCTCGGTACCATGAACCGCGTCGGGAAA | For cloning *ripU* and *ripU*_ΔNVS_ genes constructing them into the pCAMBIA 1300-nLUC vector. |
| nLUC-RipU-R | CGCGTACGAGATCTGGTCGACCTGCGGCGCGTCCTGG |  |
| 759 | GTCGCCGTCAACTCACTTTCC | *Ralstonia solanacearum*-specific primers (Opina et al., 1997) |
| 760 | GTCGCCGTCAGCAATGCGGAATCG |  |
| JG234 | GAGCGTGGTTACTCGTTCA | Reference gene (*SlActin*) of tomato in q-PCR assay (Kim et al., 2009) |
| JG136 | CTAATATCCACGTCACATTTCAT |  |
| SlJAR1-qPCR-F | GGGGGAACAGAGTATTTGAAGC | For detecting SlJAR1/ SlMYC2/ SlLOX2/SlFLS2/ SlBAK1/ SlRBOHD / SlCDPK3 mRNA levels in q-PCR assay. |
| SlJAR1-qPCR-R | CGACAATAGGGACACAAGCCT |  |
| SlMYC2-qPCR-F | ACGGAGTCGGAGAAACGATG |  |
| SlMYC2-qPCR-R | ATGATTCACGAGCACCGTCA |  |
| SlLOX2-qPCR-F | ACCATCTCAAACCCCAAGTGGA |  |
| SlLOX2-qPCR-R | CTTTCTCCAACTCCATCACCTCT |  |
| SlFLS2-qPCR-F | CTAAATGGGAGTATACCTGATAGTAT |  |
| SlFLS2-qPCR-R | GTAGGCATAAAACCAACCAAATTGT |  |
| SlBAK1-qPCR-F | GATCCTGAAGTTCACTTAGGTCAA |  |
| SlBAK1-qPCR-R | TTCCTTTAGCCGCTTAACAGCCAC |  |
| SlRBOHD-qPCR-F | GTCACCCTTGATATCCGCGATG |  |
| SlRBOHD-qPCR-R | TCAATCTCTGCTTCCTCTGCT |  |
| SlCDPK3-qPCR-F | GAAAATGAAGCTAAAAAATCACCTTTT |  |
| SlCDPK3-qPCR-R | ATGACGGGCAGGTGATTTCTTGG |  |
| **Primer name** | **Sequence (5’-3’)** | **Description** |
| cLUC-SlJAR1-F | TACGCGTCCCGGGGCGGTACCATGAAGATGATGGTGGAAAATA | For cloning *SlJAR1* gene and constructing it into the pCAMBIA 1300-cLUC vector. |
| cLUC-SlJAR1-R | ACGAAAGCTCTGCAGGTCGACTTAACAGAAAGCAGTACTGGAA |  |
| 1300FLAG- SlJAR1-F | CAGGGGCCCGGGGTCGACATGAAGATGATGGTGGAAAATA | For cloning *SlJAR1* gene and constructing it into the pSuper1300 -3×FLAG vector. |
| 1300FLAG- SlJAR1-R | TTTGTAGTCCATGGTACCACAGAAAGCAGTACTGGAATAGC |  |
| cLUC-GUS-F | tacgcgtcccggggcggtaccATGTTACGTCCTGTAGAAACCCC | For cloning *GUS* gene and constructing it into the pCAMBIA 1300-cLUC vector. |
| cLUC-GUS-R | AcgaaagctctgcaggtcgacTCATTGTTTGCCTCCCTGCT |  |
| 6P-1-RipU-F | TAAGAAGGAGATATACATATGAACCGCGTCGGGAAAT | For cloning *ripU* gene and constructing it into the pGEX-6P-1 vector. |
| 6P-1-RipU-R | GTGGTGGTGGTGGTGCTCGAGCTGCGGCGCGTCC |  |

**Reference**

**Cong, S., Li, J.-Z., Xiong, Z.-Z., and Wei, H.-L.** (2023) Diverse interactions of five core type III effectors from *Ralstonia* *solanacearum* with plants. J. Genet. Genomics **50**, 341-352.

**Kim, J.-G., Li, X., Roden, J.A., Taylor, K.W., Aakre, C.D., Su, B., et al.** (2009) *Xanthomonas* T3S effector XopN suppresses PAMP-triggered immunity and interacts with a tomato atypical receptor-like kinase and TFT1. The Plant Cell **21**, 1305-1323.

**Opina, N., F. Tavner, G. Hollway, et al.** (1997) A novel method for development of species and strain-specific DNA probes and PCR primers for identifying *Burkholderia* *solanacearum* (formerly *Pseudomonas* *solanacearum*). Asia-Pacific J. Molec. Biol. Biotechnology **5**: 19-30.

**Wei, H.-L., Zhang, W., and Collmer, A.** (2018) Modular study of the type III effector repertoire in *Pseudomonas* *syringae* pv. *tomato* DC3000 reveals a matrix of effector interplay in pathogenesis. Cell Rep. **23**, 1630-1638.

**Zhou, Z., Bi, G., and Zhou, J.** (2018) Luciferase complementation assay for protein‐protein interactions in plants. Curr. Protoc. Plant Biol. **3**, 42-50.
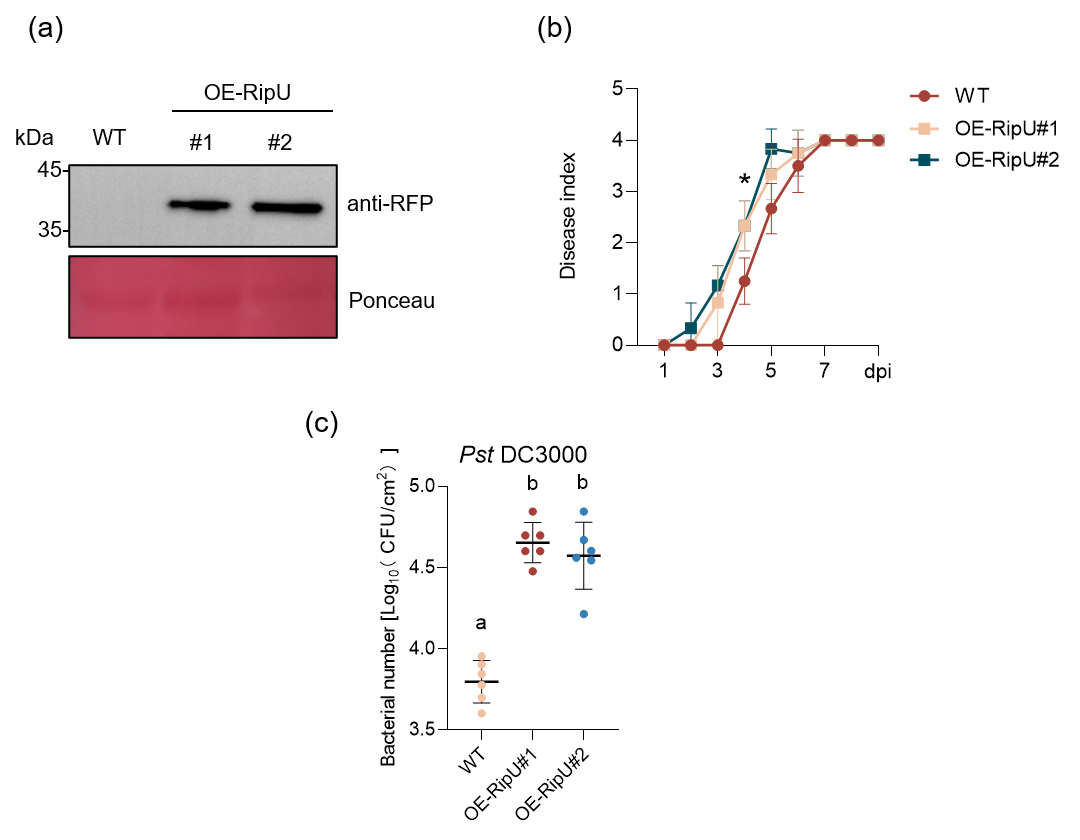


**Figure S1. Overexpression of *ripU* suppresses immune responses in tomato.** (a) RipU protein abundance in RipU-overexpression transgenic tomato was detected by immunoblot analysis with anti-RFP antibody. Total protein was extracted from 4 weeks old plants and separated by SDS-PAGE. Ponceau staining shows protein loading. (b) Soil-drenching inoculation assays in tomato plants using *R. solanacearum* P380. Disease progression was scored on a scale of 0 to 4 based on the average wilting symptoms. Values from 3 independent biological repeats were pooled together (n=10). Asterisk indicate significant differences at *P* < 0.05. (c) Growth of *Pst* DC3000 in WT and OE-RipU tomato leaves. Tomato plants were spray-inoculated with *Pst* DC3000 at 2×10^8^ CFU/mL, bacterial titers were determined at 3 dpi (n=6). All experiments were performed in three biological repeats. Different lowercase letters indicate significant differences at *P* < 0.05. Data are presented as means ± SD.


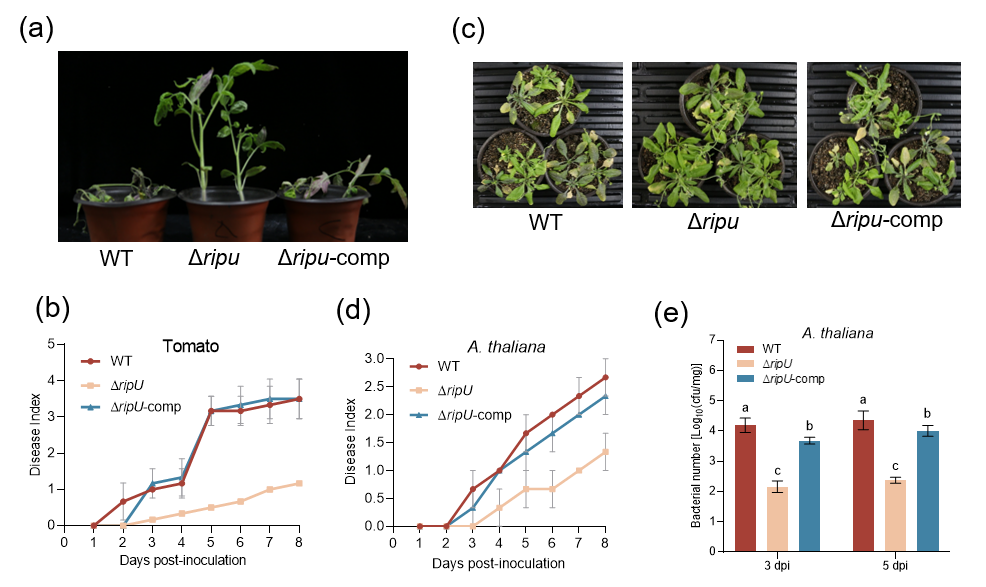


**Figure S2. RipU is critical for *R. solanacearum* virulence.** (a) and (c) Disease symptoms of WT tomato and *A*. *thaliana* plants caused by *R. solanacearum*P380 and its derivatives. Representative images were captured at 5 dpi. (b-d) Soil-drenching inoculation assays in tomato or *A. thaliana* plants using *R. solanacearum* P380 and its derivatives (10^8^ CFU/mL). Disease index was scored on a scale of 0 to 4 based on the average wilting symptoms. Values from 3 independent biological repeats were pooled together (n=12 for tomato, n=15 for *A. thaliana*). (e) Growth capacity of *R. solanacearum*P380 and its derivatives in *A. thaliana*. Bacterial load was determined at 3 and 5 dpi by plating serial dilutions of *A. thaliana* aerial parts. Different lowercase letters denote statistically significant differences (*P* < 0.05) determined by one-way ANOVA. Data are presented as means ± SD. All experiments were performed in three biological repeats.


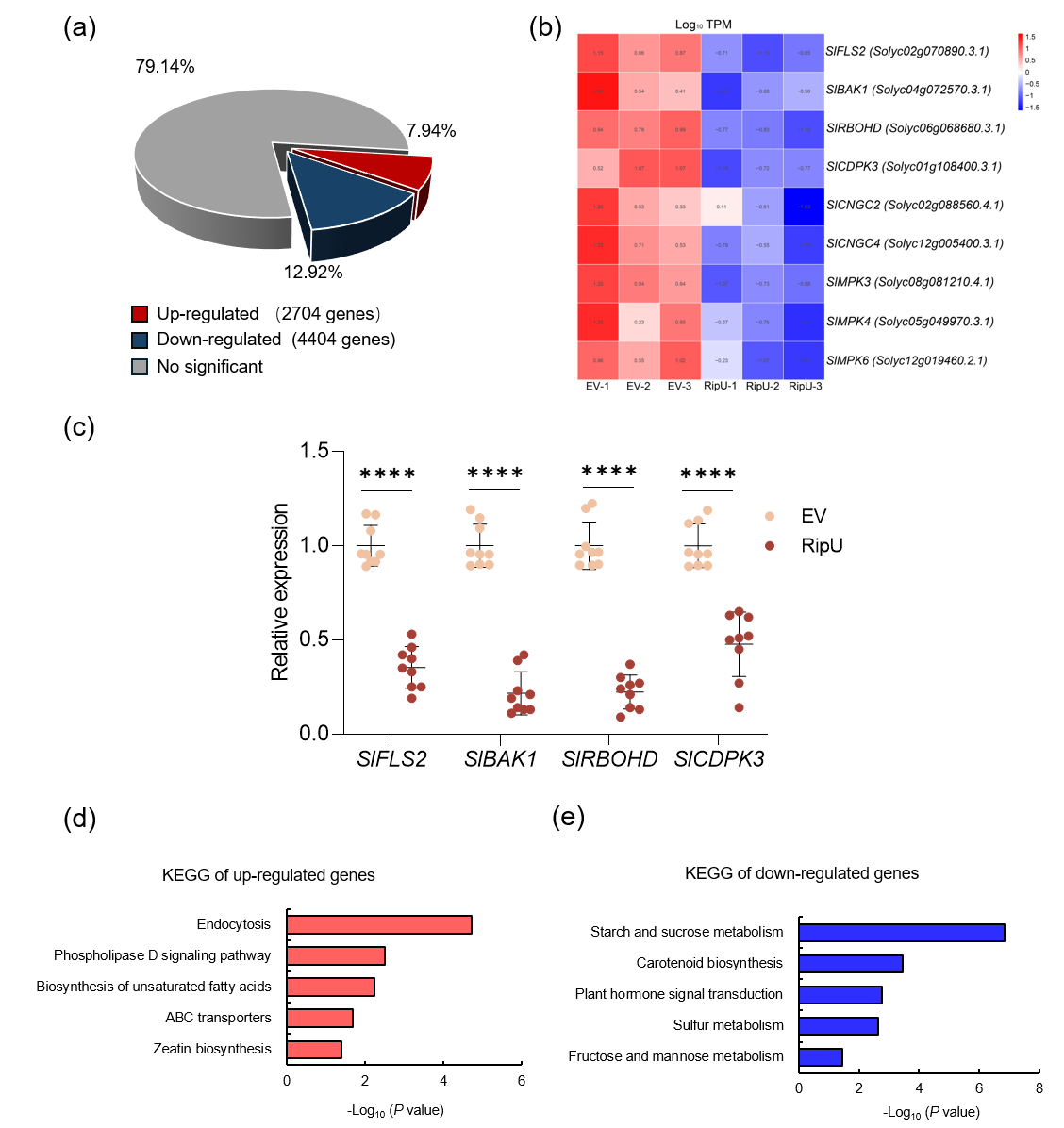


**Figure S3. Transcriptome overview of tomato leaves treated with RipU.** (a) Proportions of DEGs in the tomato genome in response to RipU. DEGs were identified using thresholds of |fold change| ≥ 2 and *P* < 0.05. Red indicates up-regulated genes, while blue indicates down-regulated genes, respectively. (b) The heatmap shows the down-regulation of defense-related genes in response to RipU. The numbers indicate Log_10_ TPM values. Gene accession numbers are indicated. (c) RT-qPCR analysis of four immunity-related genes shown in (b). D36E and its derivatives were infiltrated into 3 weeks old tomato leaves at 6 × 10^8^ CFU/mL, samples were collected at 6 hpi. Data were normalized to *SlActin* expression (n=9). Asterisks (****) represent statistical significance at *P* < 0.0001 determined by Student’s t-test. Data are presented as means ± SD. (d and e) KEGG enrichments of up- or down-regulated genes induced by RipU. Enrichment scores are shown as −Log_10_ (*P* value). All experiments were performed in three biological repeats.


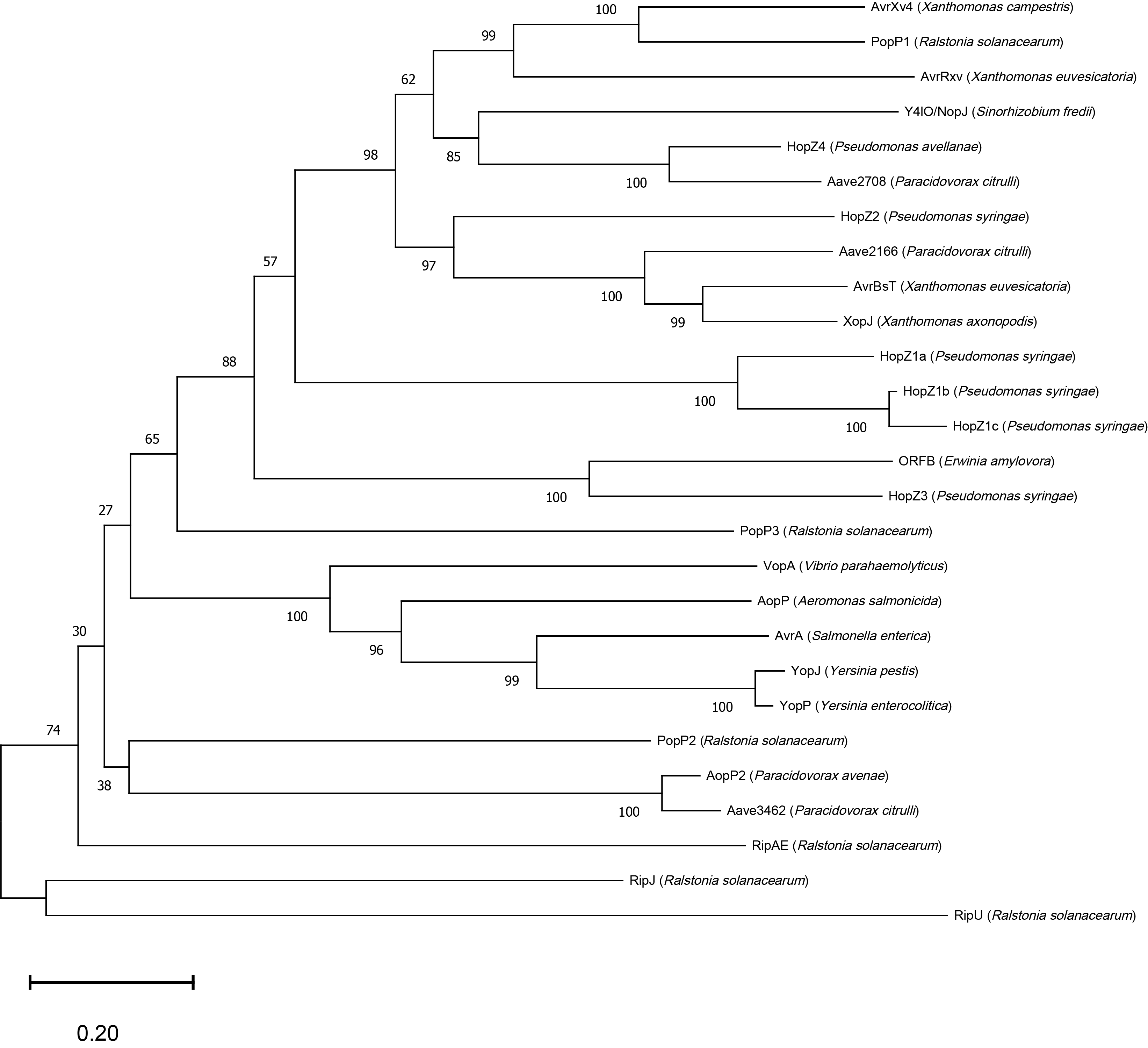


**Figure S4**. **RipU exhibits low conservation with previously reported acetyltransferase effector proteins.** Phylogenetic tree was constructed using the neighbor-joining method by MEGA 11.0.13. The evolutionary distances were computed in units of the number of amino acid substitutions per site, as shown by the scale bar.


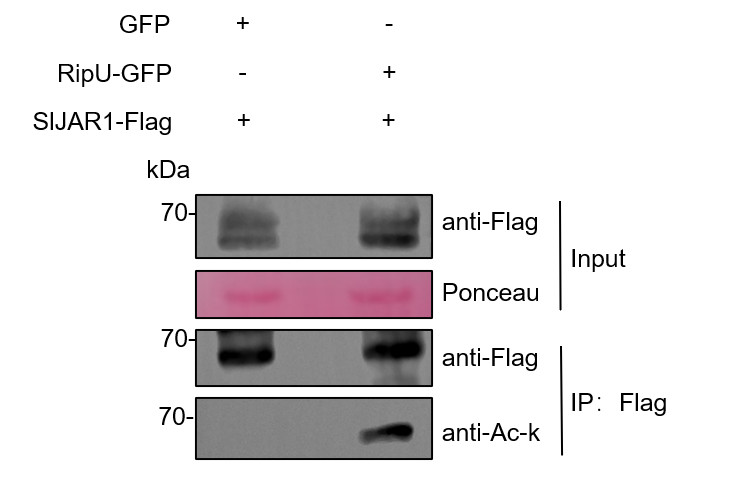


**Figure S5. RipU acetylates SlJAR1 in *N. benthamiana* leaves.** SlJAR1 was co-expressed with GFP or RipU-GFP in *N. benthsmiana* leaves. Protein extracts were immunoblotted with anti-Flag antibodies (Input). IP was conducted with anti-Flag beads and analyzed on immunoblots with anti-Flag antibody for detection of SlJAR1 or Ac‐K antibody for detection of acetylated SlJAR1. Ponceau staining shows protein loading. This experiment repeated three time with similar results.


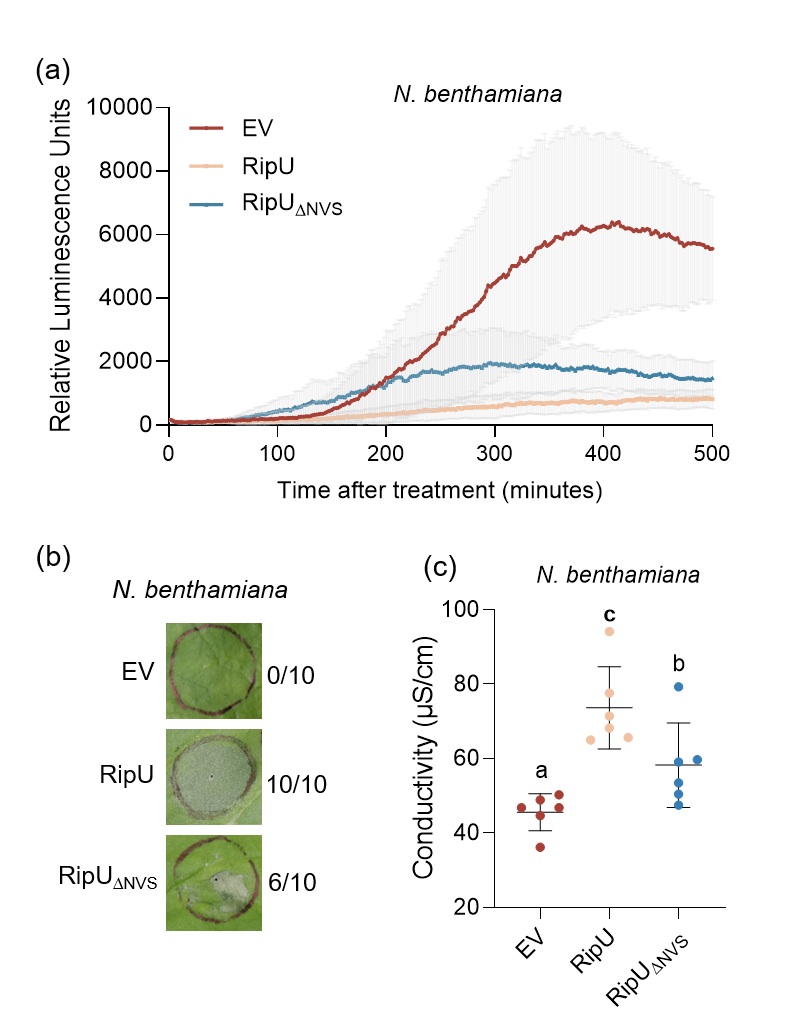


**Figure S6. The NVS residues of RipU are involved in the plant immune modulation*.*** (a) ROS burst assay. D36E and its derivatives were inoculated into 4 to 5 weeks old *N. benthamiana* leaves at 6 × 10^8^ CFU/mL. ROS production was determined at 15 h post-inoculation using L-012 chemiluminescence (n=12). (b) Cell death assay. Bacterial inoculation was carried out as above. Pictures were photographed at 3 dpi and relative numbers of collapsed inoculated zones are shown (n=10). (c) Conductivity assay. Bacterial inoculation was carried out as above, and samples were collected at 24 hpi (n=6). All experiments were performed in three biological repeats. Different lowercase letters indicate significant differences at *P* < 0.05. Data are presented as means ± SD.

**MATERIALS AND METHODS**

**Plant materials and cultivation conditions**

For bacterial inoculation assays, tomato plants (*Solanum lycopersicum* cv. Moneymaker) were cultivated at a temperature of 25°C with a 16-hour light period and 20°C with an 8-hour dark period, while maintaining a relative humidity of 40–60%. *N. benthamiana* plants were raised in soil within a greenhouse, exposed to a 16-hour light and 8-hour dark cycle at temperatures of 25°C and 22°C,and kept at 75% relative humidity, respectively. *Arabidopsis* *thaliana* plants were grown under the condition at 23°C with a 10-h-light/14-h-dark photoperiod, with a relative humidity of 60%.

**Strains, plasmids, and primers**

*Escherichia coli* was cultured in Luria-Bertani (LB) medium at 37°C. *Agrobacterium tumefaciens, Pseudomonas syringae,* and *Ralstonia solanacearum* were grown at 28°C in LB, King’s B (KB), and Nutrient Broth (NB) media, respectively. The strains, plasmids and primers used in this study are detailed in Tables above. Antibiotics were applied at the following final concentrations: ampicillin (100 μg/mL), kanamycin (50 μg/mL), rifampicin (25 μg/mL), spectinomycin (50 μg/mL), gentamicin (10 μg/mL), and chloramphenicol (10 μg/mL).

**Construction of the** P380Δ***ripU* mutant and complementation of the mutant**

An unmarked deletion *ripU* mutant was generated using a *sacB*-based homologous recombination method, as described previously (Zhang et al., 2018). Approximately 300 bp of both the upstream and downstream regions of the *ripU* gene were amplified from the genomic DNA of the *R. solanacearum* P380. The PCR fragments were ligated into pK18*mobsacB* to generate the suicide plasmid of the *ripU* gene. The resulting plasmid confirmed by sanger sequencing was transformed into P380 through electroporation. The Δ*ripU* mutant was selected through two rounds of screening and verified by PCR with primers RipU-JCF/R as detailed in Table S2. For the construction of *ripU* complemented strain, the coding sequence (CDS) of *ripU* and *ripU_ΔNVS_* genes were amplified using primers RipU-HBF/R detailed in Table S2 and inserted into pBBRMCS-2 to generate plasmid pBBRHB*ripU and* pBBRHB*ripU_ΔNVS_*. Following sequencing confirmation, the resulting plasmid was introduced into the *R. solanacearum* P380Δ*ripU* via electroporation. The successful complemented strains we verified with primers RipU-HBF/R detailed in Table S2.

**Bacterial inoculation assays**

For *R. solanacearum* soil-drenching inoculation, 4-week-old tomato or *Arabidopsis thaliana* plants were inoculated with a bacterial suspension at 10^8^ CFU/mL. The inoculated plants were incubated at 28°C with 90% humidity under 12h light/12h dark photoperiod. Disease symptom was scored on a scale of 0 to 4 (0: no wilting; 1: 1%-25% wilting; 2: 26%-50% wilting; 3: 51%-75% wilting; 4: 76%-100% wilting), and pictures were photographed at 7 post-inoculation (dpi) (Qi et al., 2022). Each experiment was replicated three times independently.

The quantification of *R. solanacearum* in tomato stems was performed as previously reported (Qi et al., 2022). Briefly, 40-mL suspension of P380 and its derivatives were poured onto the wounded roots of 4 weeks old tomato plants at 10^8^ CFU/mL, 1-cm-long tissues above stem base were collected at 3 dpi. Samples were grounded in sterile water and diluted solution was spread on NA medium with appropriate antibiotics, bacterial titers were determined after 2 days of culture at 28°C. Each experiment was replicated three times independently.

To assess the growth capacity of *R. solanacearum* in *A. thaliana*, 10 μL bacterial suspensions of the wild-type P380, Δ*ripU*, and Δ*ripU*-comp strains were inoculated onto the roots of 2-week-old *A. thaliana* plants grown on sterile MS medium at a final concentration of 1×10⁵ CFU/mL. At 3 and 5 dpi, the entire aerial parts of the seedlings were collected and thoroughly homogenized using sterile grinding equipment. The number of single colonies was then enumerated.

OE-RipU and WT tomato plants were spray-inoculated with *Pst* DC3000 suspensions at 2×10^8^ CFU/mL. Inoculated tomato plants were placed under a plastic cover to maintain high humidity. Leaf samples were collected at 3 dpi for bacterial growth determination. Each experiment was replicated three times independently.

**ROS burst assay**

Bacterial suspensions of D36E-EV, D36E-RipU, and D36E-RipU_∆NVS_ were infiltrated into 5-week-old tomato leaves at 6 × 10^8^ CFU/mL. At 6 hours post-inoculation (hpi), leaf disks (each 0.25 cm²) were collected and placed into the wells of a 96-well plate containing 10 µL of sterile water. For ROS measurement of *N. benthamiana* plants, leaf disks were harvested at 15 hpi. Subsequently, 100 µL of 0.5 mM L-012 (Japan Wako, Cat: WTP7511) in 10 mM morpholinepropanesulfonic acid–KOH buffer (pH 7.4) was added to each well. ROS production was then monitored in real-time using a Multimode Reader Platform (Tecan, Switzerland). Each experiment was replicated three times independently.

The flg22-induced ROS production was assessed as previously reported with minor modifications (Sang and Macho, 2017). **L**eaf disks were punched from OE-RipU and WT tomato leaves and placed into 96-well plates containing 100 μL of sterile water, followed by incubation for 12 hours. The water was then removed, and a reaction mixture containing 100 nM flg22 (GenScript, Cat: RP19986CN), 100 μM luminol (Sigma, Cat: A8511), and 20 μg/ml horseradish peroxidase (Sigma, Cat: P8375-5KU) was added to each well. ROS production was monitored in real-time using a Multimode Reader Platform (Tecan, Switzerland) for 1 hour, with readings taken every 2 minutes. Each experiment was replicated three times independently.

**Assays for cell death and conductivity**

D36E-EV, D36E-RipU, and D36E-RipU_∆NVS_ were infiltrated into 5-week-old tomato leaves at 10^8^ CFU/mL . At 12 hpi, the leaves were photographed and the number of necrotic lesions was counted. For conductivity measurements, bacterial suspension were infiltrated into 5-week-old tomato leaves at 6×10^7^ CFU/mL, leaf disks (1 cm in diameter) were collected from the infiltrated areas at 12 hpi and submerged in 3.5 mL of sterile deionized water. The leaf samples were incubated at 37°C with shaking at 220 rpm for 3 hours, and the conductivity of ion leakage into the water was measured using a conductivity meter (Leici, China). The assays described above in *N. bethamiana* plants were performed as previously reported (Cong et al., 2023). Each experiment was replicated three times independently.

**Generation of transgenic tomato plants**

pGWB554-RipU was transformed into *A. tumefaciens* GV3101 and was then transformed into tomato plants as previously described (Girhepuje et al., 2011). To examine the presence of RipU, leaves of OE-RipU and WT tomato were collected and subject to total protein extraction using the buffer (50 mM Tris-HCl pH 7.5, 150 mM NaCl, 0.1% NP-40, 4 M urea, 1 mM PMSF). Then the protein samples were subjected to immunoblot analysis using anti-RFP mouse monoclonal primary antibody (Abbkine, Cat: AE020) at a dilution of 1:2500 and HRP - conjugated goat anti-Mouse IgG secondary antibody at a dilution of 1:5000 (Abbkine, Cat: A21010). Each experiment was independently replicated three times.

**RNA sequencing**

Fresh D36E-EV and D36E-RipU were collected, resuspended in 10mM MgCl_2_ to a final concentration at 6 × 10^8^ CFU/mL, then infiltrated into 3-week-old tomato leaves, and maintained in the greenhouse for 6 hours before sampling. Three biological replicates were analyzed for each treatment. RNA sequencing was performed by Majorbio (Shanghai, China) on an Illumina HiSeq instrument according to manufacturer’s instructions (Illumina, San Diego, CA, USA). Quality control (QC) was implemented with FASTP v 0.23.4 on the raw sequencing data before proceeding to alignment and downstream analyses. HISAT2 v2.2.1 was utilized for aligning RNA-seq reads to the reference genome of *Solanum lycopersicum* genome (Heinz1706) with the default parameters (Kim et al., 2015). After alignment, the number of mapped reads per sample was derived and normalized to Transcripts Per Kilobase of exon model per Million mapped reads (TPM) (Li et al., 2011). DEGs were identified using the DESeq2 v1.42.0 package based on the raw count data using thresholds of |fold change| ≥ 2 and *P* < 0.05 (Love et al., 2014). The raw sequencing data have been deposited in the NCBI under accession number PRJNA1287065.

**Reverse transcription quantitative PCR**

To determine the expression level of JA related genes, D36E-EV and D36E-RipU were infiltrated into 3-week-old tomato leaves at 6 × 10^8^ CFU/mL. Leaf samples were harvested at 6 hpi. Total RNA was isolated from the infiltrated tomato leaves using TRIzol reagent following the manufacturer’s protocol (Beijing Cwbio Biotech, Cat: CW580S). Subsequently, reverse transcription quantitative PCR (RT-qPCR) was conducted using a Reverse Transcription Kit (Biosharp, Cat: BL696A) and SGExcel Fast SYBR Master Mix (Shanghai Sangon Biotech, Cat: B532995). The primers for RT-qPCR are detailed in Table S2. Gene expression levels were determined by the 2−^ΔΔ^Ct method (Livak and Schmittgen, 2001) and normalized against *SlActin* (Kim et al., 2009). Each experiment was replicated three times independently.

**Hormone content determination assay**

To determine whether RipU regulates the JA content of tomato plants, bacterial suspensions of D36E-RipU and D36E-EV were infiltrated into 4-week-old tomato leaves at 6 × 10^7^ CFU/mL. The leaf samples were collected at 24 hpi and subjected to JA content measurement by a phytojasmonic acid enzyme-linked immunoassay kit according to the manufacturer’s instructions (Jonlnbio, Cat: JL13627). To examine whether RipU modulates tomato JA content during *R. solanacearum* infection, 3-week-old tomato plants were dip-inoculated into the bacterial suspensions of P380, Δ*ripU*, and Δ*ripU-*comp at 10^8^ CFU/mL for 48 h. To investigate whether overexpressing *ripU* alters the JA-lle content, the roots of 3-week-old WT and OE-RipU tomato plants were collected and subjected to further analysis. JA-lle contents were detected by MetWare (http://www.metware.cn/)based on the AB

Sciex QTRAP 6500 LC-MS/MS platform.

**Split-LUC assay**

**Split-LUC assay was performed as previously described** (Zhou et al., 2018)**. *A. tumefaciens* strains were incubated in the infiltration buffer (10 mM MgCl_2_, 10 mM MES, 200 mM acetosyringone, pH 5.6) for 3 h, and adjusted to** 5 × 10^8^ CFU/mL **for each strain. *A. tumefaciens* strains containing the desired plasmids were infiltrated into 4-week-old *N. benthamiana* leaves using a blunt-end syringe. Split-LUC assay was performed at 3 dpi. For qualitative analysis, the leaves were infiltrated with 0.5 mM luciferin (Solarbio, Cat: D8390) in water and kept in the dark for 5 min before CCD imaging with IVIS Lumina LT system (PE, USA).**

**Co-IP**

Co-IP assay was performed as previously described (Wang et al., 2021). ***A. tumefaciens* strains** harboring the corresponding plasmids were infiltrated into 4-week-old *N. benthamiana* leaves at 5 × 10^8^ CFU/mL, and leaf samples were collected at 48 hpi. The samples were treated with Native Buffer [50 mM Tris–MES, pH 8.0, 1 mM NaCl, 10 mM EDTA, 0.5 M Sucrose, and protease inhibitor cocktail (CWBIO, Cat: CW2200S)]. The homogenate was thoroughly mixed and incubated on ice for 10 minutes. After centrifugation at 15,000 × g for 10 minutes at 4 °C, the supernatant was collected. A 100 μL aliquot of the supernatant was removed, mixed with SDS-PAGE loading buffer (Solarbio, Cat: P1015), boiled at 95 °C for 10 minutes, and used as the input protein. The remaining supernatant was incubated with FLAG agarose (Selleck, Cat: B23101) or GFP agarose (Tiandirenhe, Cat: SA070005) at 4°C for 4 hours. The agarose beads were then pelleted by centrifugation at 500 × g for 2 minutes at 4°C and washed three times with pre-cooled Native Buffer. SDS-PAGE buffer was added to the beads, which were boiled at 95°C for 10 minutes to elute the bound proteins (output). Both input and output proteins were analyzed by immunoblotting using anti-FLAG (Abbkine, Cat: A02010) or anti-GFP (ABclonal, Cat: AE012) antibodies. Each experiment was replicated three times independently.

**Acetylation assay**

For the autoacetylation activity assay, purified RipU protein was subjected to

immunoblot analysis using anti-His (MBL, Cat: D291-7) and anti-acetyl-lysine (PTM Bio, Cat: PTM-105RM) antibodies. RipU digested with proteinase K (Biosharp, BL104B; 37°C, 10 min) and PBS were controls. Each experiment was independently replicated three times.

*In vitro* acetylation assay was performed as previously reported (Huang et al., 2025). Briefly, SlJAR1-Flag-His was co-expressed with either RipU-GST or RipU_∆NVS_-GST in *E. coli* BL21. Recombinant proteins were purified using BeaverBeads™ His-Tag Purification Kit (BEAVER, Cat: 70521P-100) according to the manufacturer’s protocol.

Acetylated lysine residues were detected with anti-acetyl-lysine antibody (PTM Bio, Cat: PTM-105RM). Protein expression was verified by immunoblotting using anti-FLAG antibody (Abbkine, Cat: A02010) and anti-GST antibody (MBL, Cat: PM013-7). Total protein loading was normalized by Coomassie Brilliant Blue (CBB) staining. Each experiment was independently replicated three times.

*In vivo* acetylation assay was performed as previously described (Le Roux et

al., 2015). Briefly, *A. tumefaciens* strains containing the desired plasmids were

infiltrated into 4-week-old *N. benthamiana* leaves using a blunt-end syringe at 5 × 10^8^ CFU/mL. Leaf samples were harvested at 48 hpi and subjected to protein extraction.

FLAG-tagged proteins were immunoprecipitated with anti-DYKDDDDK Tag (Bimake,

Cat: B23102), followed by immunoblotting with anti-acetyllysine antibody (PTM Bio, Cat: PTM-105RM) and anti-FLAG antibody (Abbkine, Cat: A02010), respectively. Each experiment was independently replicated three times.

**Bioinformatic analysis**

GO analysis was performed using the topGO v2.36.0 package with default settings under the Rv3.6.0 environment. KEGG pathways analysis was performed using R2HTML v2.3.3 and an online KEGG database. Significant GO and KEGG terms were enriched by setting the *P* < 0.05. The heatmap was generated using the online toolbox (<https://www.genescloud.cn/chart/HeatMap>). The tertiary structure of the RipU protein was predicted using the AlphaFold3 website (https://alphafold.ebi.ac.

uk/).

**Statistical analysis**

All experiments were performed in three biological repeats. Statistical analyses were performed using SPSS 17.0 (SPSS, Inc., Chicago, IL, USA) and GraphPad Prism 8.0 (GraphPad, Inc., La Jolla, CA, USA). Significance was assessed using Student’s t-test or one-way ANOVA followed by Tukey’s post hoc test. Data are presented as means ± SD. Results with *P*-values less than 0.05 were deemed statistically significant.

**Accession numbers**

The sequence of RipU can be found in the genome of *Ralstonia solanacearum* P380 (GenBank accession number: JAKEEA000000000). The corresponding SGN number for SlJAR1 is Solyc10g011660.2. The raw RNA-seq data are available in the NCBI database under BioProject accession number PRJNA1287065. The amino acid sequences used in this study for phylogenetic analysis were sourced from a published work (Ma and Ma, 2016).

**Reference**

**Sang Y., Macho, A. P.** (2017) Analysis of PAMP-Triggered ROS burst in plant immunity. Methods Mol. Biol. **1578**,143-153.

**Cong, S., Li, J.-Z., Xiong, Z.-Z., and Wei, H.-L.** (2023) Diverse interactions of five core type III effectors from *Ralstonia* *solanacearum* with plants. J. Genet. Genomics **50**, 341–352.

**Huang, J., He, P., Zhong C., Qin, T., Wang, H., Shi J., et al.** (2025) Bacterial acetyltransferase effector AopP2 primes effector-triggered immunity in watermelon by acetylating a conserved transcription factor. Plant Cell Environ. **48**, 8650-8667.

**Girhepuje, P.V. and Shinde, G.B.** (2011) Transgenic tomato plants expressing a wheat endochitinase gene demonstrate enhanced resistance to *Fusarium* *oxysporum* f. sp. *lycopersici*. Plant Cell Tiss. Organ Cult. **105**, 243–251.

**Kim, D., Langmead, B., and Salzberg, S.L.** (2015) HISAT: a fast spliced aligner with low memory requirements. Nat. Methods **12**, 357–360.

**Kim, J.-G., Li, X., Roden, J.A., Taylor, K.W., Aakre, C.D., Su, B., et al.** (2009) *Xanthomonas* T3S effector XopN suppresses PAMP-triggered immunity and interacts with a tomato atypical receptor-like kinase and TFT1. The Plant Cell **21**, 1305–1323.

**Le Roux, C., Huet, G., Jauneau, A., Camborde, L., Trémousaygue, D., Kraut, A., et al.** (2015) A receptor pair with an integrated decoy converts pathogen disabling of transcription factors to immunity. Cell **161**, 1074–1088.

**Li, B. and Dewey, C.N.** (2011) RSEM: accurate transcript quantification from RNA-Seq data with or without a reference genome. BMC Bioinformatics, **12**, 323

**Livak, K.J. and Schmittgen, T.D.** (2001) Analysis of relative gene expression data using real-time quantitative PCR and the 2^−ΔΔCT^ method. Methods **25**, 402–408.

**Love, M.I., Huber, W., and Anders, S.** (2014) Moderated estimation of fold change and dispersion for RNA-seq data with DESeq2. Genome Biol. **15**, 550

**Ma, K.-W., Ma W.** (2016) YopJ family effectors promote bacterial infection through a unique acetyltransferase activity. Microbiol. Mol. Biol. Rev. **80**,1011-1027

**Qi, P., Huang, M., Hu, X., Zhang, Y., Wang, Y., Li, P., et al.** (2022) A *Ralstonia* *solanacearum* effector targets TGA transcription factors to subvert salicylic acid signaling. The Plant Cell **34**, 1666–1683.

**Wang, S., Li, S., Wang, J., Li, Q., Xin, X.-F., Zhou, S., et al.** (2021) A bacterial kinase phosphorylates OSK1 to suppress stomatal immunity in rice. Nat. Commun. **12**, 5479

**Zhang, X., Zhao, M., Yan, J., Yang, L., Yang, Y., Guan, W., et al.** (2018) Involvement of *hrpX* and *hrpG* in the virulence of *Acidovorax* *citrulli* strain Aac5, causal agent of bacterial fruit blotch in cucurbits. Front. Microbiol. **9**, 507

**Zhou, Z., Bi, G., and Zhou, J.** (2018) Luciferase complementation assay for protein‐protein interactions in plants. Curr. Protoc. Plant Biol. **3**, 42–50.
